# APBSmem: A Graphical Interface for Electrostatic Calculations at the Membrane

**DOI:** 10.1371/journal.pone.0012722

**Published:** 2010-09-29

**Authors:** Keith M. Callenberg, Om P. Choudhary, Gabriel L. de Forest, David W. Gohara, Nathan A. Baker, Michael Grabe

**Affiliations:** 1 Carnegie Mellon-University of Pittsburgh Program in Computational Biology, University of Pittsburgh, Pittsburgh, Pennsylvania, United States of America; 2 Department of Biological Sciences, University of Pittsburgh, Pittsburgh, Pennsylvania, United States of America; 3 The Edward A. Doisy Department of Biochemistry and Molecular Biology, Saint Louis University, St. Louis, Missouri, United States of America; 4 Pacific Northwest National Laboratory, Richland, Washington, United States of America; 5 Department of Computational and Systems Biology, School of Medicine, University of Pittsburgh, Pittsburgh, Pennsylvania, United States of America; University of Oxford, United Kingdom

## Abstract

Electrostatic forces are one of the primary determinants of molecular interactions. They help guide the folding of proteins, increase the binding of one protein to another and facilitate protein-DNA and protein-ligand binding. A popular method for computing the electrostatic properties of biological systems is to numerically solve the Poisson-Boltzmann (PB) equation, and there are several easy-to-use software packages available that solve the PB equation for soluble proteins. Here we present a freely available program, called *APBSmem*, for carrying out these calculations in the presence of a membrane. The Adaptive Poisson-Boltzmann Solver (APBS) is used as a back-end for solving the PB equation, and a Java-based graphical user interface (GUI) coordinates a set of routines that introduce the influence of the membrane, determine its placement relative to the protein, and set the membrane potential. The software Jmol is embedded in the GUI to visualize the protein inserted in the membrane before the calculation and the electrostatic potential after completing the computation. We expect that the ease with which the GUI allows one to carry out these calculations will make this software a useful resource for experimenters and computational researchers alike. Three examples of membrane protein electrostatic calculations are carried out to illustrate how to use APBSmem and to highlight the different quantities of interest that can be calculated.

## Introduction

The relationship between the electric field and the charge in a system is determined by Maxwell's equations; however, several factors contribute to making these equations difficult to solve in a heterogeneous, condensed phase. The most popular method for carrying out electrostatic calculations in a biological setting is to solve the Poisson-Boltzmann (PB) equation. Starting from a known protein structure, this method treats the protein and water as distinct dielectric environments, and the charges on the protein give rise to the electric field. Additionally, PB theory implicitly accounts for counter-ions in solution via a non-linear term that depends on the bulk counter-ion concentration and the electrostatic potential. The PB equation for a one-to-one electrolyte solution is:
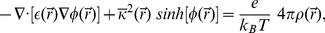
(1)where 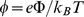 is the reduced electrostatic potential and 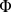 is the electrostatic potential, 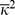 is the Debye-Hückel screening parameter, which accounts for ionic shielding, 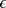 is the dielectric constant for each of the distinct microscopic regimes in the system, and 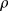 is the density of charge within the protein moiety. Since the 1980s, researchers have studied the electrostatic properties of protein and nucleic acid systems by numerically solving the PB equation using finite difference and finite element methods [1]–[4]. Today, there are several popular software packages available to perform PB calculations such as DelPhi [5], the Adaptive Poisson-Boltzmann Solver (APBS) [6], MIBPB [7], ZAP [8], and the PBEQ module in CHARMM [9]. Unfortunately, there is a dearth of programs that allow researchers to carry out these calculations at or near a membrane. Nonetheless, over the last two decades the number of high-resolution membrane protein structures has dramatically increased. The membrane has several unique electrical properties. For instance, the core of the membrane is extremely hydrophobic giving rise to a desolvation penalty for moving charged molecules into this region. This property is essential to the membrane's ability to control the flow of materials into and out of the cell. Additionally, most cells have a substantial membrane potential that coordinates the action of voltage-dependent membrane proteins such as voltage-gated ion channels. Without including the effects of the membrane dielectric and the transmembrane potential, there is a huge class of molecules whose electrical properties cannot easily be explored.

Groups have carried out simulations using several different levels of theory to include the effects of the membrane such as fully atomistic calculations (for an incomplete list of references see [10]–[16]), implicit membrane calculations using Generalized Born models (for an incomplete list see [17]–[29]), and continuum approaches employing the PB equation (for an incomplete list see [30]–[41]); however, all of these studies require the user to have a high level of computational sophistication as highlighted by the relatively few papers from non-computational laboratories. Thus, it is desirable to have a program that removes many of the technical and bookkeeping aspects from these calculations. Toward this end, very recently, an online web server was created to facilitate PB calculations on membrane proteins using the PBEQ module [42], and here, we present our program, APBSmem, which combines an easy to use interface with APBS to allow non-experts to calculate the electrostatic properties of membrane proteins. APBSmem can be downloaded, easily installed, and run locally on Windows, Mac, and Linux platforms. APBSmem has several pull down templates that allow researchers to carry out specific membrane related calculations, and it has a built-in graphical window that provides quick visual feedback to make sure that the system is set up correctly and to view results.

## Methods

### User interface

**Figure 1:**
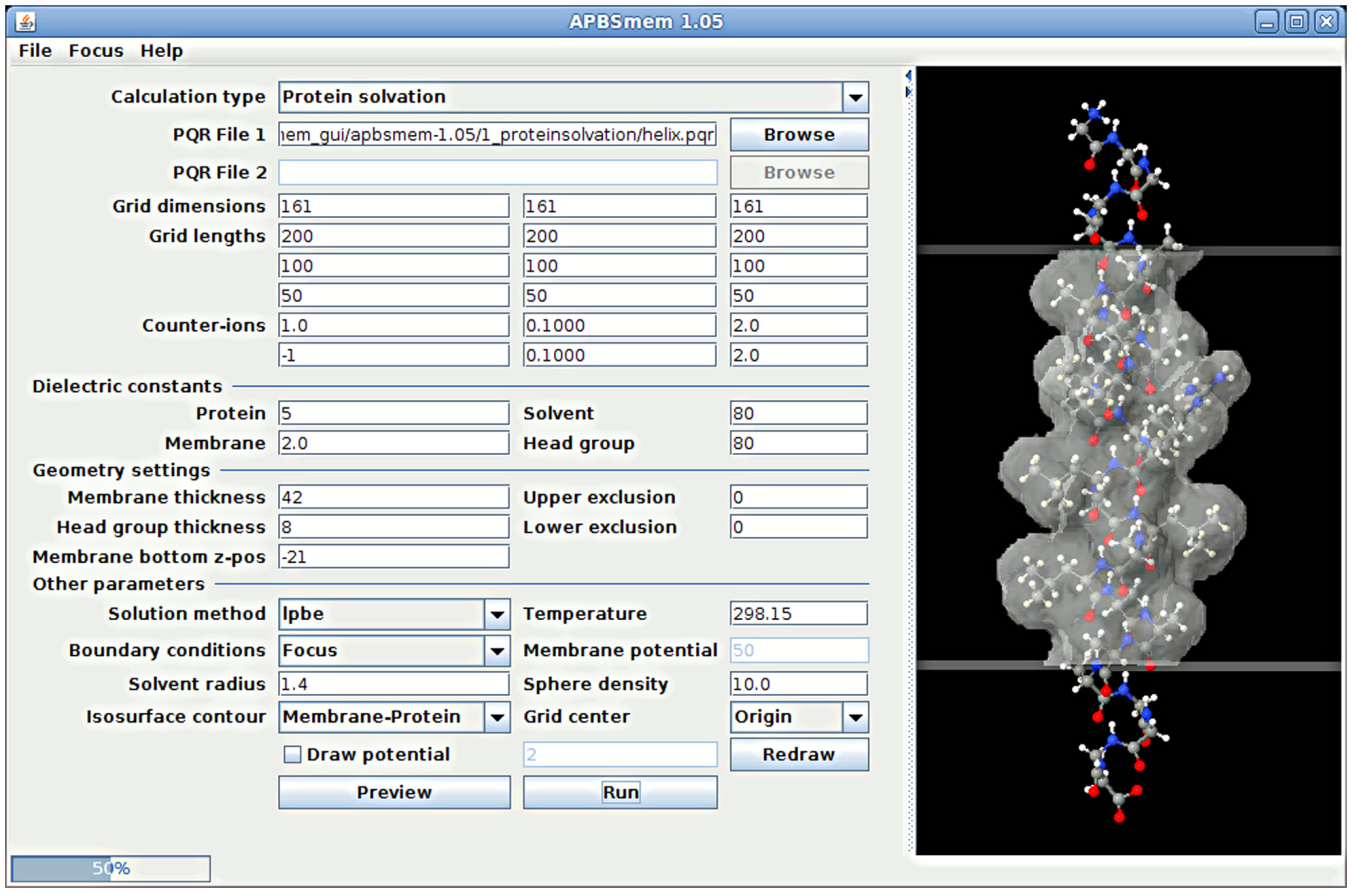
A screenshot of the user interface. Parameters pertaining to the calculation are entered in the field on the left, and the molecule and membrane can be viewed in the embedded Jmol viewer on the right. Pictured here is the membrane-embedded single transmembrane helix used for the calculations in CASE I.

Though the majority of the calculations described here may be performed using APBS input files, keeping track of the files and parameters can become quite difficult. To improve this process we built a Java-based GUI that writes the input files and runs the calculations (Figure 1). The GUI has an embedded Jmol [43] viewer that allows users to visualize the protein-membrane system and the electrostatic potential. Here we explain the necessary parameters and use of the interface in a step-by-step fashion.

#### Calculation Type

To perform a calculation, the user first selects a type (Protein Solvation, Ion Solvation, or Gating Charge) from the drop down menu. Each type is described in more detail in the case studies below. The user then selects coordinate files, in PQR format, for all of the protein configurations of interest. PQR files contain the atomic positions of all of the atoms in the system in addition to their charge and radii. PQR files can be generated from PDB files with the PDB2PQR tool [44], which allows the user to choose from several commonly used charge and radii parameter sets: PARSE [45], CHARMM27 [46], Swanson [47], AMBER99 [48], along with several other sets and user defined values. This choice is crucial since calculations can be sensitive to the parameter set [49] especially when performing solvation energy calculations. File locations may be entered manually or found and selected from the filesystem with the Browse button. At present, APBSmem does not allow the spatial orientation and placement of the protein to be altered once read in through the GUI, and external software must be employed if a different orientation is desired. For Protein Solvation calculations, the user should provide a coordinate file with only the membrane protein. Ion Solvation calculations require a PQR file with only the protein and a PQR file with only the ion. Two files corresponding to an open and a closed channel are needed in the case of a Gating Charge calculation.

#### Grid Spacing

Next, the user must specify the desired level of discretization, which is related to the fidelity with which the underlying equations will be solved. It is necessary to apply the appropriate boundary conditions far from the protein to accurately solve electrostatics calculations, and this requires large grid lengths to remain computationally tractable. However, coarse discretization does not capture the correct electrostatic behavior near the protein, where small grid spacing is needed. A technique known as focusing is employed to rectify this problem by solving the equations in a series of steps starting at the largest length scale and focusing into the smallest length scale [5]. When using multiple levels of focusing, the user can set the desired level in the Focus menu to enable fields for additional grid lengths.

**Figure 2:**
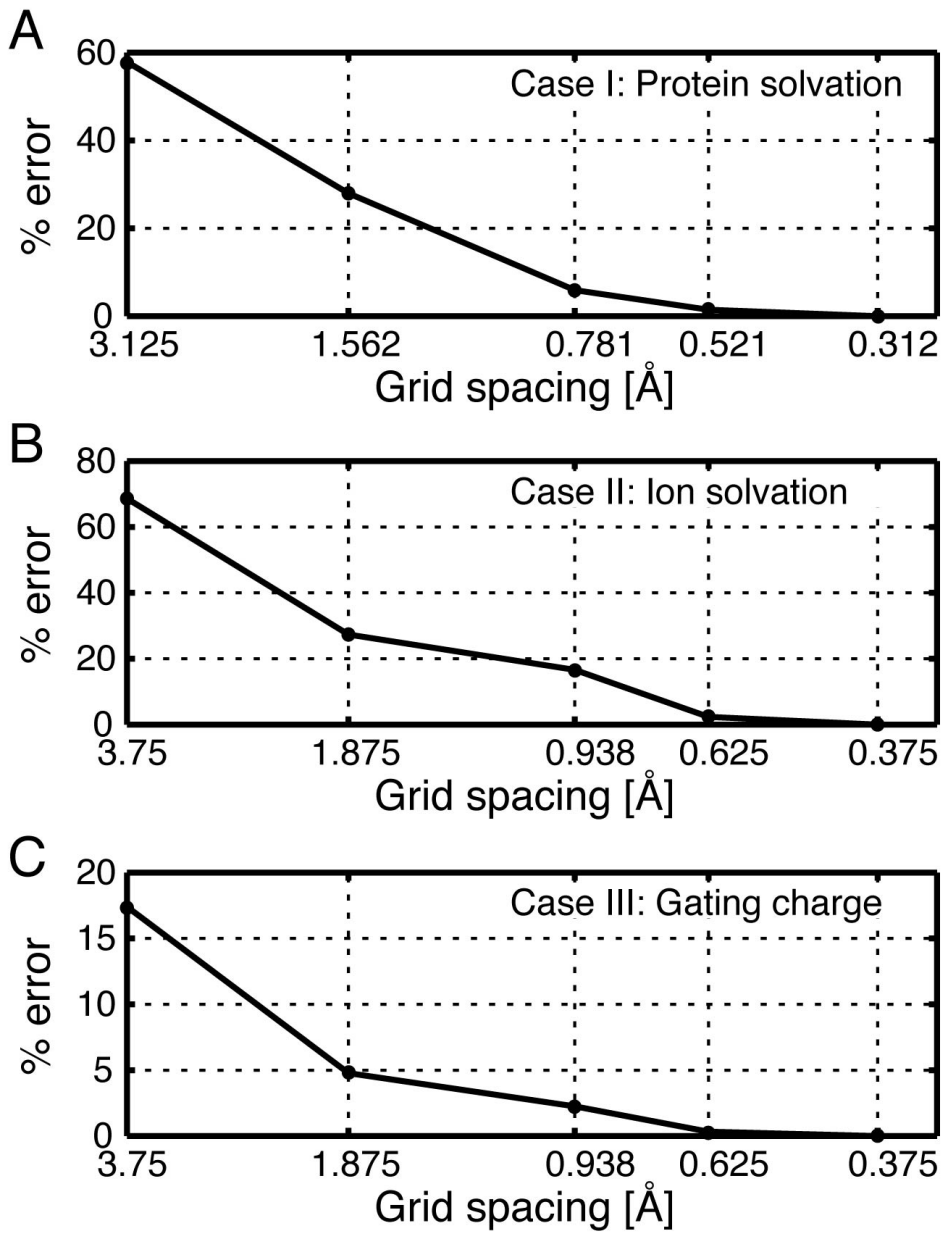
Convergence properties of test cases I–III. We computed the absolute value of the percent error, 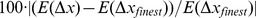, for each test case using a number of different discretization values. All energies are reported with respect to the solution value at the finest level of discretization, 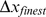, which was 0.312 Å in test Case I and 0.375 Å in Cases II and III. Values along the x-axis are spaced using a log base 2 scale. In all graphs, the number of grid points used to achieve the grid spacing on the x-axis was 17, 33, 65, 97, 129, and 161 (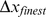). (A) Convergence of the protein solvation energy, Case I. A grid spacing of 0.512 Å gives a solution 1.5% of the highest resolution value. The energy values smoothly converge as the resolution increases. (B) Convergence of the ion solvation energy, Case II. The error monotonically decreases as the level of discretization increases. At 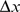 = 0.625 Å the energy value is within 2.5% of the final value. (C) Convergence of the gating charge energy in the closed state, Case III. Rather than report the gating charge, here we plot the energy of the closed state. This method converges much more quickly than the other Cases since it does not involve Born Self energy terms. The energy at the second finest level is 0.33% of the value at the finest level. Even at a grid spacing of 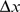 = 0.938 Å the computed energy is within 3% of the best value. In all cases, the convergence properties and the accuracy of the solutions depend critically on the refinement of the protein surface boundaries. Here we use the spl2 method for charge mapping in APBS, which gives very desirable convergence properties.

With any numeric calculation, the accuracy of the solution is directly related to the degree of discretization. It is important to check the convergence properties of your solution. This is typically done by recalculating with increasing numbers of grid points without changing any of the other parameters. The exact convergence properties depend on the numeric algorithm and the details of the system. In Figure 2, we calculated the convergence for each test case in the Results section. However, all systems behave differently, and users should not assume that discretization schemes that give accurate results here will also give accurate values for other protein-membrane systems.

#### Dielectric Parameters

In continuum electrostatics, different regions of the system are defined by unique dielectric values. These values are related to the polarizability of each region in response to an applied electric field. The protein dielectric value is assigned to points within the protein's solvent accessible surface. All points outside the protein, but within the membrane region defined by the geometry settings, are assigned a dielectric value corresponding to the membrane. While the core of the membrane often has a very low dielectric value, the head group region may be characterized by a much higher value. If desired, this physical feature can be included in calculations by increasing the default head group thickness and setting the head group dielectric value. All other points in the system are assigned the solvent dielectric value.

Proteins are heterogeneous, and it is not always appropriate to describe the entire molecule with a single uniform dielectric value [50]. Nonetheless, uniformity is a common assumption of PB solvers. Experiments indicate that the protein interior is modeled best by dielectric values between 2 and 20 [51]. With this in mind, we recommend that researchers test the robustness of their results by repeating calculations with several different dielectric constant values within this range.

#### Boundary Conditions

Several options are offered for Dirichlet boundary conditions when solving the PB equation in APBS. The user may set all boundaries to zero, use a single Debye-Hückel model, multiple Debye-Hückel model, or focusing, in which the boundaries are determined by a previous calculation. When the Gating Charge calculation type is chosen, the boundary condition is set to impose a range of membrane potentials across the membrane as described in Case III. The user provides a membrane potential value in milliVolts, 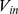, and the interface performs a sweep of calculations from 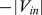 to 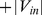 to determine the valence of the gating motion. At present, calculations with a membrane potential are only carried out in the linearized limit of Eq. 1. Additionally, application of the boundary conditions ignores differences between the dielectric values of the head group and the membrane core, which has been included in a recent study [52].

#### Protein Surface Representation

An accurate representation of the protein surface is important in constructing the dielectric and ion-accessibility maps. A probe-based dielectric function is used to construct the protein surface in APBS. The solvent probe radius specifies the size of water spheres for the determination of the solvent space and is typically set to 1.4 Å for water. The surface sphere density determines the resolution at which this boundary is calculated and is typically set to 10 grid points/Å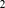.

#### System Geometry

**Figure 3:**
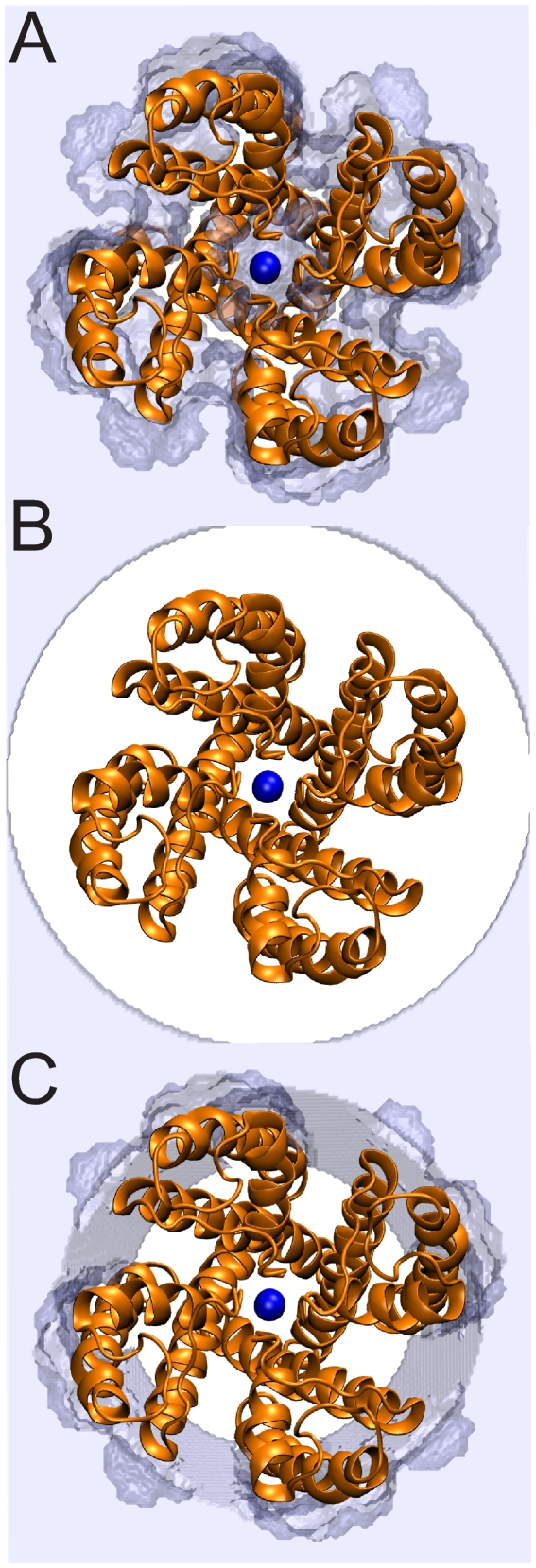
Top view of the KcsA channel (green) and the 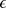 = 2.01 isocontour highlighting the membrane interface (gray). The K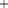 ion in the center of the channel is shown in blue. (A) When the membrane is not excluded from the channel pore, we observe that membrane is added to the pore region. (B) With the exclusion radii set too high at 28 Å, there are large gaps of water between the outer membrane and the protein. (C) The channel should be clear of membrane and the membrane should fit snugly around the outside of the protein as shown here. Membrane exclusion radii are 24 Å and 16 Å at the top and bottom of the channel, respectively.

The system geometry parameters determine the shape and location of the membrane. The membrane thickness and vertical position should be adjusted for the protein of interest so that the bilayer interfaces with the protein as expected. It is difficult to determine this placement, and it is an ongoing area of research in the Grabe lab [53]. In practice, this placement is often done ad hoc based on the location of the hydrophobic residues making up the membrane spanning region. A better alternative is to first estimate the orientation of the membrane protein and extent of the membrane spanning region by using the Orientations of Proteins in Membranes database [54]. A second point of concern is that ion channels and transporters often have hydrophilic, water-filled cavities essential for transport. Users must employ the interface exclusion radii to prevent APBSmem from rewriting these cavities as membrane. These radii should be adjusted so that central cavities, if present, are filled with water and the membrane is arranged flush with the outside of the protein. We intend to provide automated cavity detection in future releases. Figure 3 compares correct and incorrect configurations of the membrane geometry with the KcsA potassium channel.

#### System Preview

After the user has entered parameters for the dielectric environment and membrane geometry, the Preview button can be used to visualize the system. This Preview action performs a quick “dummy” calculation with coarse grid dimensions to generate the numeric representation of the membrane and display it graphically. If the system and parameters appear to be correct, the user can click Run to perform the calculation with APBS. When the calculations have completed, the total energy is given in kJ/mol, kcal/mol and k
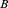
T, and the most focused dielectric map of the membrane is displayed in the Jmol panel. The electrostatic potential may also be viewed in the Jmol panel by selecting the Draw Potential option. The user provides an isocontour value of interest and the interface displays the positive (red) and negative (blue) surfaces over the protein. The exterior bulk and cavity (if any) at the interior of the protein are modeled into APBS as coefficient maps (openDX-format). These maps include dielectric maps (diel), ion-accessibility coefficient maps (kappa) and charge distribution maps (charge) for different regions of the protein-membrane complex. All input and output files, including the potential and DX maps are saved for later use and reference.

### Membrane potential boundary conditions

In a typical cell, electrogenic transporters create a difference between the electrical potential inside the cell, 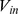, and outside the cell, 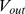. A small violation in electroneutrality near the membrane gives rise to this potential difference; however, more than a Debye length from the membrane, electroneutrality is restored. It is possible to model this behavior with the Poisson-Boltzmann equation as outlined by Roux in his seminal work on this topic [55]. Most researchers will want to compute the membrane potential in the absence of protein charges to understand the profile across the protein, and in some cases, they will be interested in computing the interaction energy of the membrane electric field with the charges on the protein. The field due to the protein charges and the membrane potential are only separable when solving the linearized form of the equation. Thus, in order to be self consistent, APBSmem only solves the linearized Poisson-Boltzmann equation when employing membrane potential boundary conditions. In future releases, we will extend this to the full non-linear equation. The presentation in this section and the next largely follows the supporting text found in Grabe *et al.* [38], but the essence is similar to Roux's [55]. We start by rewriting Eq. 1 in the linearized form:
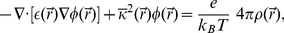
(2)


**Figure 4:**
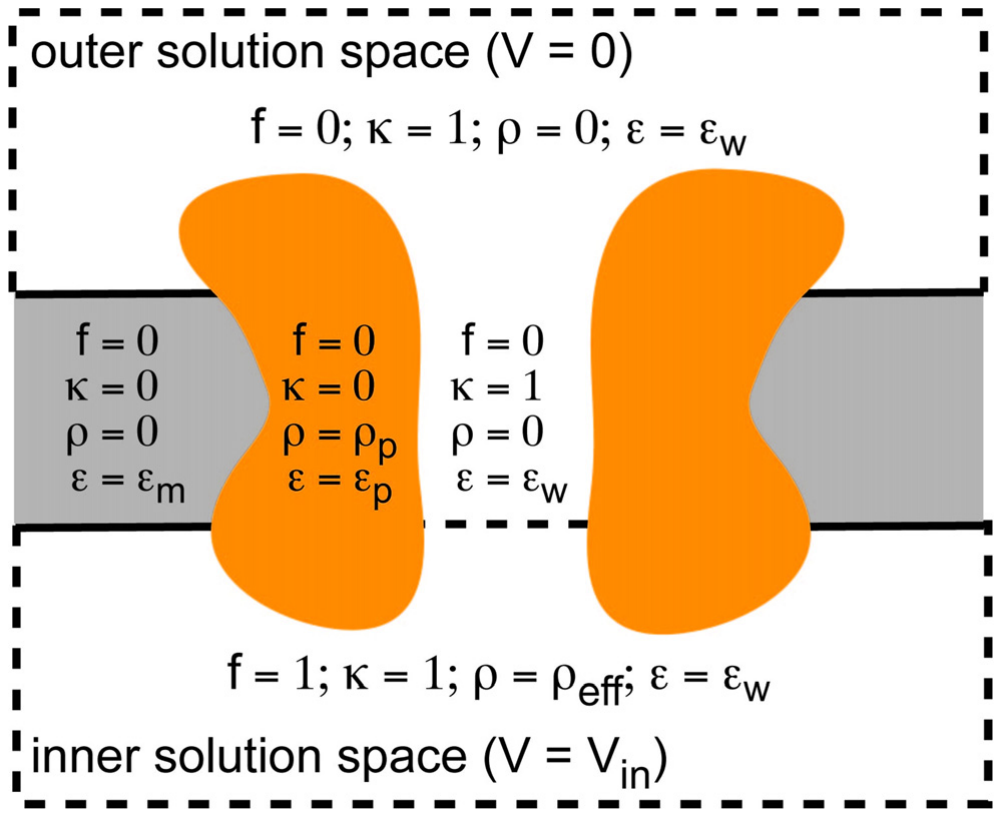
A cartoon representation of the distinct dielectric environments in each calculation. The orange regions represent protein, the gray membrane, and all white areas indicate water. The inner solution space at the bottom is assigned a voltage of 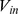, and correspondingly an effective charge density 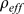 is assigned and a value of one for the variable 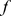. The water in the center of the channel is assigned values for 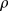 and 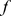 that correspond to the outer solution space. The lower 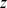 value of the membrane (dashed line) separates the inner and outer solution spaces. In the gray region, 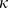 and 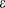 are set to 0 and 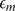, respectively, to mimic the membrane.

However, this equation does not satisfy the asymptotic boundary condition: 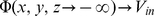. This oversight can be corrected by adding a constant term to the equation for all positions in the inner solution space:
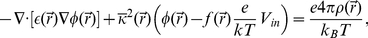
(3)where 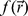 is 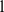 for all points in the inner solution space and zero otherwise (see Figure 4). Now far from the protein where 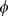 is no longer changing, 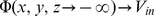 as desired. Eq. 3 can be rewritten as:
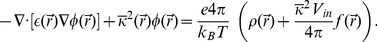
(4)


Thus, the modified Poisson-Boltzmann equation above takes the form of Eq. 2 with the membrane potential arising from a term that looks like a uniform source charge. The spatial dependence of 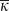 is carried by 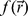 on the right hand side. Since Eq. 4 is linear, it is possible to separate the total reduced electrostatic potential, 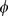, into contributions from the membrane potential, 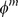, and contributions from the protein, 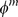, as 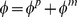. Each field is the solution to corresponding equations as shown:
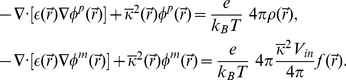
(5)


Far away from the protein, 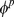 approaches zero. Poisson-Boltzmann solvers typically set zero boundary conditions at the outer boundary to account for this behavior, or they use some asymptotic approximation to the field based on the protein's total charge. In the case of a membrane potential, the behavior of 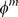 far from the membrane protein is required so that far field boundary conditions can be imposed on the system.


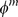
 on the boundary is determined by considering a planar slab of low-dielectric material with symmetric electrolyte solution in the half-spaces above and below the slab. This follows the work of Roux with a slight change in geometry [55]. By symmetry 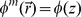, and we assign 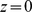 to the center of the membrane. The slab has a length 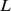, and there are three distinct regions of space: 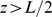 (out); 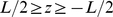 (membrane); 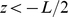 (in). The dielectric of water is 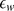, and the dielectric constant of the membrane is assigned 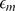. We assume that ions cannot enter the membrane so 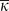 is set to 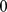 in this region, while the inner and outer spaces have the same value of the screening parameter. According to Eq. 5 the 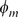 satisfies the following equations in each region
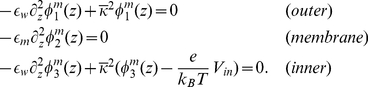
(6)


From elementary electrostatics, we know that the potential is continuous at the membrane boundaries but the z-component of the electric field is discontinuous due to the jump in dielectric value:
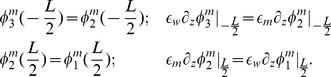
(7)


The potential profile can be determined from Eqs. 6 and 7:
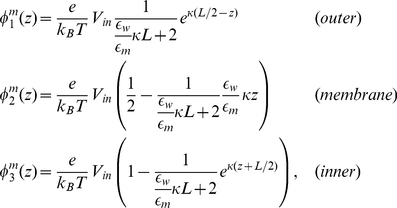
(8)where 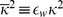. When membrane potential calculations are performed, Eq. 8 is used to set 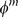 on the domain boundary. This requires first providing the z-position of the top and bottom of the membrane and the dielectric constants of the membrane and water.

### Addition of the membrane

The influence of the membrane must be included in the calculation. Based on the structure file provided, the program calls on APBS to generate dielectric (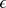), charge (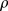), and ion-accessibility maps (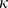) of the molecule as if it were in solution. The protein dielectric value can be set to any value, and the method for delineating the solvent boundary is also configurable. At present, the GUI allows up to 2 levels of focusing, which corresponds to 3 sets of maps produced at this initial stage. Maps are then modified to add the presence of a low-dielectric slab acting as a surrogate membrane. APBS is run with the finite differencing scheme option; therefore, all map points are associated with a regular grid in 3-space. Next, the initial maps are read by a second routine, and the numeric values of points on the grid are modified based on the spatial position and the user-defined placement of the membrane. The program iterates over every grid position and evaluates each position in the following order:

#### 1) Determine if the point is inside the provided protein

If the initial dielectric map value equals 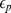, the point is located within the protein. Dielectric map values are not changed for these points.

#### 2) Determine if the point is inside the membrane

If the point does not fall within the protein, it falls within the 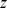-extent of the membrane determined by 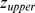 and 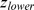, and it falls outside the cylinder described by the exclusion radii, the value of the dielectric map is set to 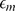, the ion-accessibility is set to zero, and the charge map is not changed.

#### 3) Determine if the point is in the inner solution space

If the point is below the membrane and the ion-accessibility is not zero, then the neutral charge map is modified for the calculations of 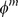. The value assigned to the charge map position is determined from Eq. 5 (bottom equation). The effective charge density, 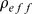, follows from the right hand sides of the upper and lower equations:
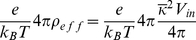



The text maps are written in terms of the number density, 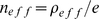, and using this along with the definition of the Debye length we arrive at the modified value for the charge map
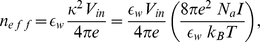
where 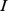 is the molar concentration of one of the salt species (assumed balanced) and 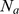 is Avogodro's number. The Debye constant above is twice the value that can be found on page 497 of Jackson's Classical Electrodynamics (Second Edition) [56], since we assume that there are mobile cationic and anionic species, not just one mobile species. Simplifying this equation we arrive at:
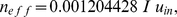
where 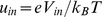 is the reduced inner potential and the counter-ion concentration is given in moles per liter. The effective number density is now in inverse Å ngstroms cubed, which is consistent with the APBS solver.

## Results

APBSmem was developed in Java and requires Java Runtime Environment 5.0 or later and APBS version 1.2.0 or later which can be downloaded from http://java.sun.com/ and http://www.poissonboltzmann.org/, respectively. The program can be run from the command-line using java -jar apbsmem.jar. Three case studies are presented here to demonstrate potential calculations. All files necessary to perform these calculations are packaged with the APBSmem program.

### CASE I: Protein Solvation

**Figure 5:**
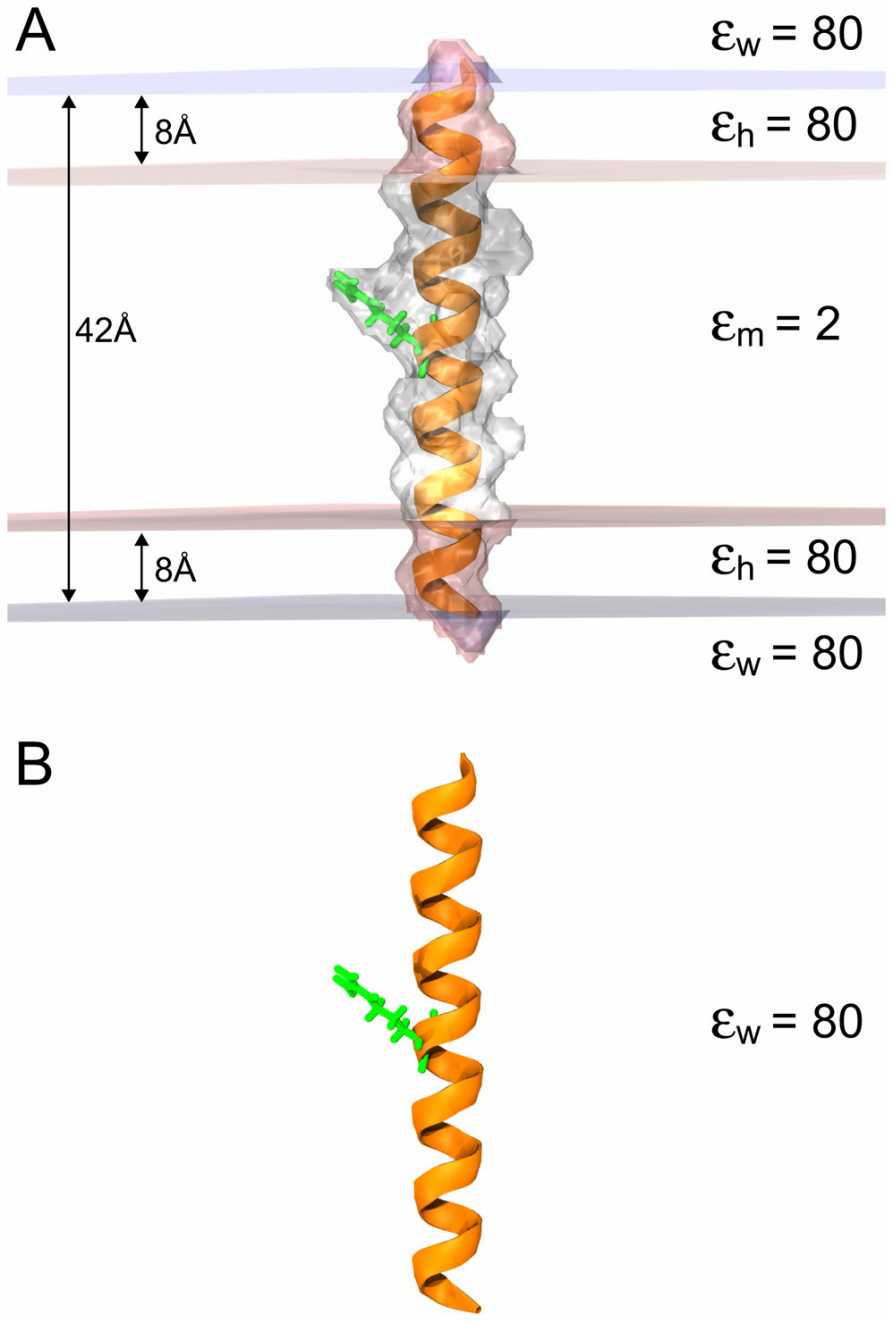
States used to compute protein solvation energies. (A) The helix (orange) is pictured embedded in the membrane, which is delineated by the upper blue and lower gray lines. The membrane core between the two red lines is assigned a dielectric value 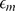 = 2. A headgroup region of 8 Å is indicated between the water and membrane core. Bulk water above and below the membrane is assigned a dielectric value of 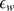 = 80. (B) The helix in the bulk water (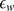 = 80) in the absence of the membrane. The helix carries one charged residue (Arg14) shown in green in (A) and (B). The protein solvation energy is calculated by computing the total electrostatic energy of systems A and B and then calculating the quantity: 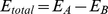. Images rendered with VMD [63].

The cell membrane is composed of lipid molecules and hosts membrane proteins which account for a third of all proteins in a cell. The hydrophobic core of the membrane provides a dielectric barrier against polar and charged molecules. The transmembrane segments of membrane proteins are therefore largely composed of hydrophobic residues; but charged and polar residues are also sometimes present, so it is natural to ask how these charged residues can be stably accomodated in the membrane. Choe *et al.* [53] investigated this question using continuum electrostatics with APBS. Here we revisit this problem to demonstrate the applicability of our graphical interface, and we do this by calculating the solvation energy required to insert a charged helix into the membrane. The total energy of a simple 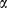-helix in bulk water (Figure 5B) is first computed and then subtracted from the total energy of the helix embedded in the membrane (Figure 5A).

Using APBSmem to compute the protein solvation energy requires the protein to be read in as PQR file 1. The system of interest for this case study is an 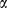-helix composed of 27 residues, aligned along the z-axis and centered at the origin. The helix is composed of nonpolar hydrophobic residues with the exception of a charged arginine at the center. The protein solvation energy calculation is performed on a 161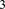 grid using two levels of focusing from a cube with side length 200 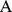 to a cube of side length 50 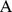. The bathing solution contains 0.1 M symmetric monovalent salt with 2 Å probe radii. The protein is assigned a dielectric value of 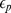 = 5, bulk water is assigned a value of 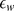 = 80, and membrane is assigned a dielectric of 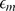 = 2. The head group is modeled as a region of high dielectric, 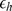 = 80, with a thickness of 8 Å. Calculations are carried out with the linearized PB equation with a solvent probe radius of 1.4 Å, a surface sphere density of 10 gridpoints/Å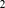 and a temperature of 298.15 K. The total membrane thickness is 42 Å running from z = −21 Å to z = +21 Å. The upper and lower exclusion radii are set to 0 Å since there is no pore. Parameters are summarized in Table 1.

**Table 1:** Parameters for protein solvation CASE I.

Parameter	Value
Calculation type	Protein solvation
PQR File	Helix.pqr
Grid Dimensions	161 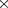 161 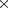 161
Coarse Grid Lengths	200 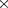 200 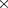 200
Medium Grid Lengths	100 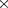 100 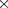 100
Fine Grid Length	50 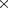 50 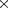 50
Counter-Ions	1.0, 0.10, 2.0
	−1.0, 0.10, 2.0
Protein Dielectric	5.0
Solvent dielectric	80.0
Membrane Dielectric	2.0
Headgroup Dielectric	80.0
Solution Method	lpbe
Boundary Condition	Focus
Solvent probe radius (srad)	1.4
Surface sphere density (sdens)	10.0
Temperature	298.15
Z-position of membrane bottom	−21
Membrane thickness	42
Head group thickness	8
Upper exclusion radius	0
Lower exclusion radius	0

For this system, we obtain a protein solvation energy of 28 kcal/mol, and Figure 2A indicates good convergence with grid spacing smaller than 0.781 Å at the finest level. While this energy is large, it is greatly reduced when non-polar energies are considered. Additionally, a large component of this energy is the cost of inserting the charged arginine. If the arginine is replaced with an alanine, the solvation energy drops to 4 kcal/mol. It has been shown that the electrostatic component of the membrane deformation energy can be considerably reduced by allowing the membrane to bend around the charged residue in the core of the membrane [53]. We will incorporate membrane bending and non-polar energy terms in future releases of APBSmem.

### CASE II: Ion Solvation

The primary role of ion channels is to facilitate the movement of ions across the dielectric barrier imposed by the lipid bilayer. The hydrated ions in the bulk water are essentially stripped of water molecules (depending on the channel pore size) upon entering a low-dielectric medium [57]–[59]. The total ion solvation free energy of an ion consists of a Born solvation term, which corresponds to the removal of water molecules away from the ion and an electrostatic term that corresponds to interaction between protein charges and the ion. APBSmem calculates the ion solvation free energy by first computing the total energy of the protein-ion assembly embedded in the membrane and then subtracting the energies of the membrane-embedded protein without the ion and the energy of the ion in bulk water.

Roux and MacKinnon carried out a classic study using this approach to investigate the transfer energy for a single K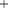 from bulk water to the central cavity of the potassium channel KcsA [60]. Here we revisit this calculation. KcsA (PDB ID 1BL8) is aligned along the z-axis and centered at the origin. The ion solvation calculation requires: a PQR file with only the KcsA ion channel and a PQR file consisting of a K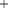 ion at the coordinate of interest. The ion transfer free energy is calculated using a finite difference method on a 161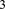 grid with two levels of focusing from a cubic system of side length 300 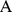 to a cube of side length 60 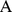. The bathing solution contains 0.1 M symmetric monovalent salt with 2 Å probe radii. The protein is assigned a dielectric interior of 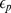 = 2, bulk water above and below the membrane, a dielectric of 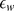 = 80, and a low-dielectric slab of dielectric value 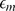 = 2 represents the membrane. The separate dielectric for the head group region (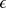 = 80) is not used since its thickness is set to zero. The linearized PB equation is solved using focused boundary conditions (one level of focusing) at 298.15 K in the absence of membrane potential. The solvent probe radius is set to 1.4 Å and a surface sphere density of 10 gridpoints/Å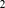 is used. The z-position of the bottom of the membrane and thickness of the membrane slab are set to −12 Å and 24 Å, respectively. Membrane exclusion radii of 24 Å and 16 Å are used for the channel at the top and bottom, respectively (Figure 3C). Parameters are summarized in Table 2.

**Table 2:** Parameters for ion solvation free energy CASE II.

Parameter	Value
Calculation type	Ion solvation
PQR file 1	KcsA PQR file
PQR file 2	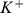 ion PQR file
Grid dimensions	161 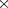 161 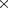 161
Coarse grid lengths	300 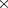 300 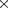 300
Medium grid lengths	120 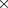 120 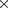 120
Fine grid length	60 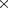 60 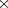 60
Counter-ions	1.0, 0.03, 2.0
	−1.0, 0.03, 2.0
Protein dielectric	2.0
Solvent dielectric	80.0
Membrane dielectric	2.0
Headgroup dielectric	80.0
Solution method	lpbe
Boundary condition	Focus
Solvent probe radius (srad)	1.4
Surface sphere density (sdens)	10.0
Temperature	298.15
Z-position of membrane bottom	−12
Membrane thickness	24
Headgroup thickness	0
Upper exclusion radius	24
Lower exclusion radius	16

**Figure 6:**
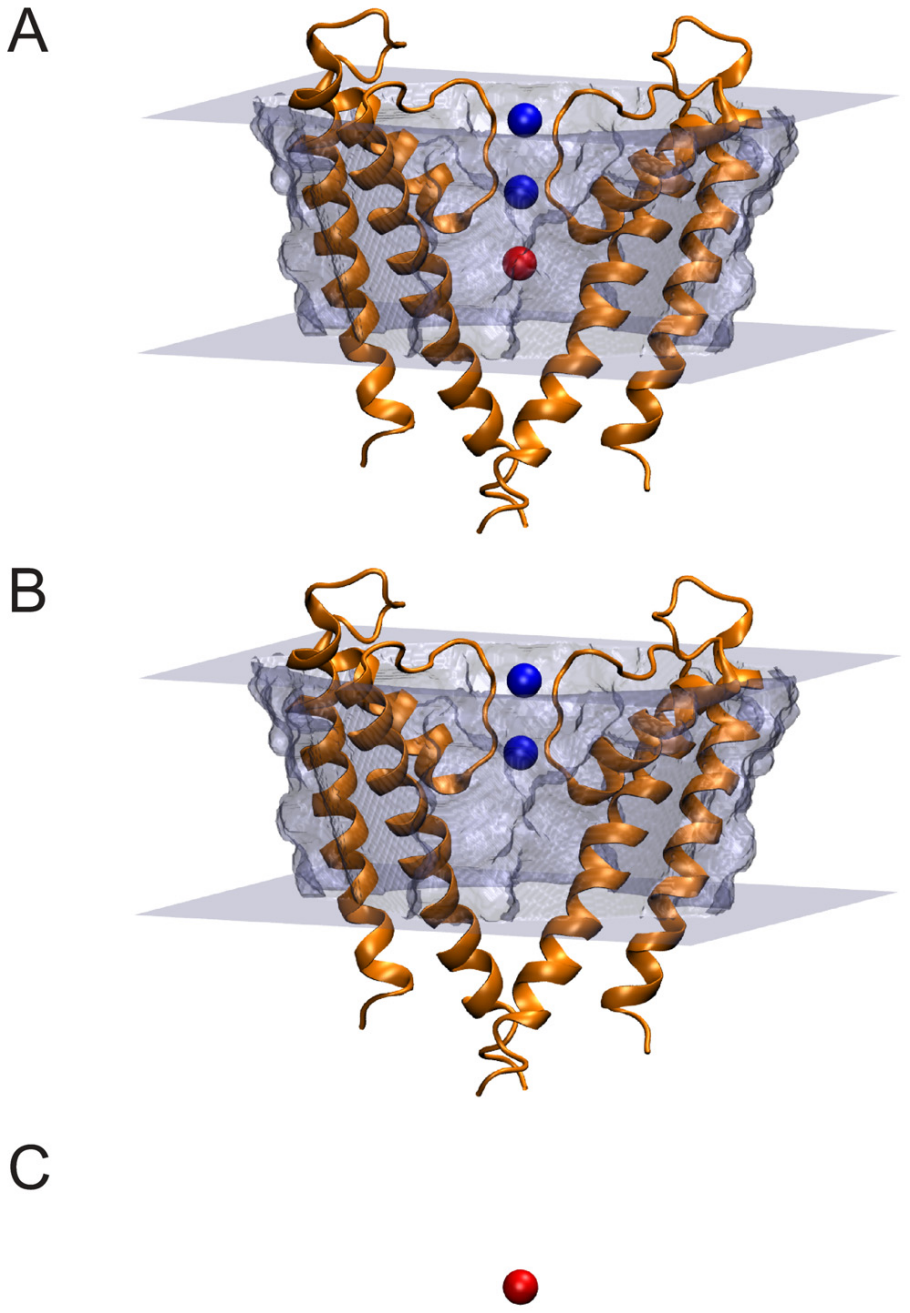
States used to compute ion solvation energies. (A) KcsA ion channel (orange) embedded in a slab of low-dielectric material (gray) with two ions in the selectivity filter (blue) and a single ion in the water filled cavity (red). For clarity only two subunits are shown. (B) Set up in panel A without the cavity ion. (C) The cavity ion in bulk water in the absence of KcsA and the membrane. The ion solvation energy is calculated by computing the total electrostatic energy of each system in A, B and C and then calculating the quantity: 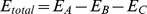.

APBSmem performs nine calculations: three sequential focusing calculations on the protein-ion system embedded in the membrane (Figure 6A), three sequential focusing calculations on just the protein in the membrane (Figure 6B) and three sequential focusing calculations on the K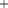 ion in bulk water (Figure 6C). Note that the system in Figure 6C computes the self energy of K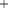 in bulk water. APBSmem obtains the ion solvation energy by subtracting the energy values obtained from the fine grid calculation of systems in Figure 6B and 6C from the system in Figure 6A, and a grid spacing of 0.625 Å or smaller gives well converged values (Figure 2B).

Using these parameters, the calculated transfer free energy (from bulk water to the center of the cavity) is 7.5 kcal/mol for a single K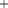 ion when protein charges are turned off. When two K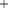 ions (blue spheres in Figure 6) are present in the selectivity filter, the calculated transfer free energy increases to 16.2 kcal/mol. This is due to electrostatic repulsion between the K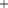 ions in the selectivity filter and the incoming K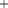 ion. Upon turning protein charges on and in the presence of two K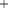 ions in the selectivity filter, the transfer free energy drops to −8.3 kcal/mol. Four pore helices (residues 62–74) along with the two 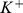 ions in the selectivity filter account for an ion transfer free energy of −3.5 kcal/mol. While there are minor differences between some of our calculated values and those of Roux and MacKinnon (see Table 3), we believe that the same conclusions can be drawn from our values.

**Table 3:** Ion solvation free energy (kcal/mol).

	Roux and MacKinnon [60]	Calculated values
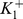 only	6.3	7.5
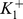 , 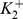 , 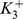 only	16.3	16.2
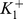 , 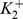 , 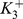 and all protein	−8.5	−8.3
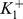 , 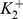 , 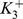 and pore helices only	−4.5	−3.5

### CASE III: Gating Charge

Voltage-gated ion channels are sensitive to changes in membrane potential. The charged residues of the channel experience a force due to the electric field across the membrane-channel complex, and this force drives the channel to open and closed conformations as the membrane potential changes. The voltage dependence of conformational changes can be described by an equivalent “gating charge” or “sensor valence” that is defined as the fraction of the membrane electric field traversed by charges on the protein during the gating process. Thus, a gating charge of 1 indicates that a unit charge has moved through the entire membrane electric field. The gating charge often adopts non-integer values, and the higher the gating charge of a channel, the steeper its voltage dependence. The theory for using continuum electrostatic calculations to determine sensor valence was developed previously [55]. Briefly, the modified PB equation considers the transmembrane potential and calculates the interaction energy of protein charges with the field.

**Figure 7:**
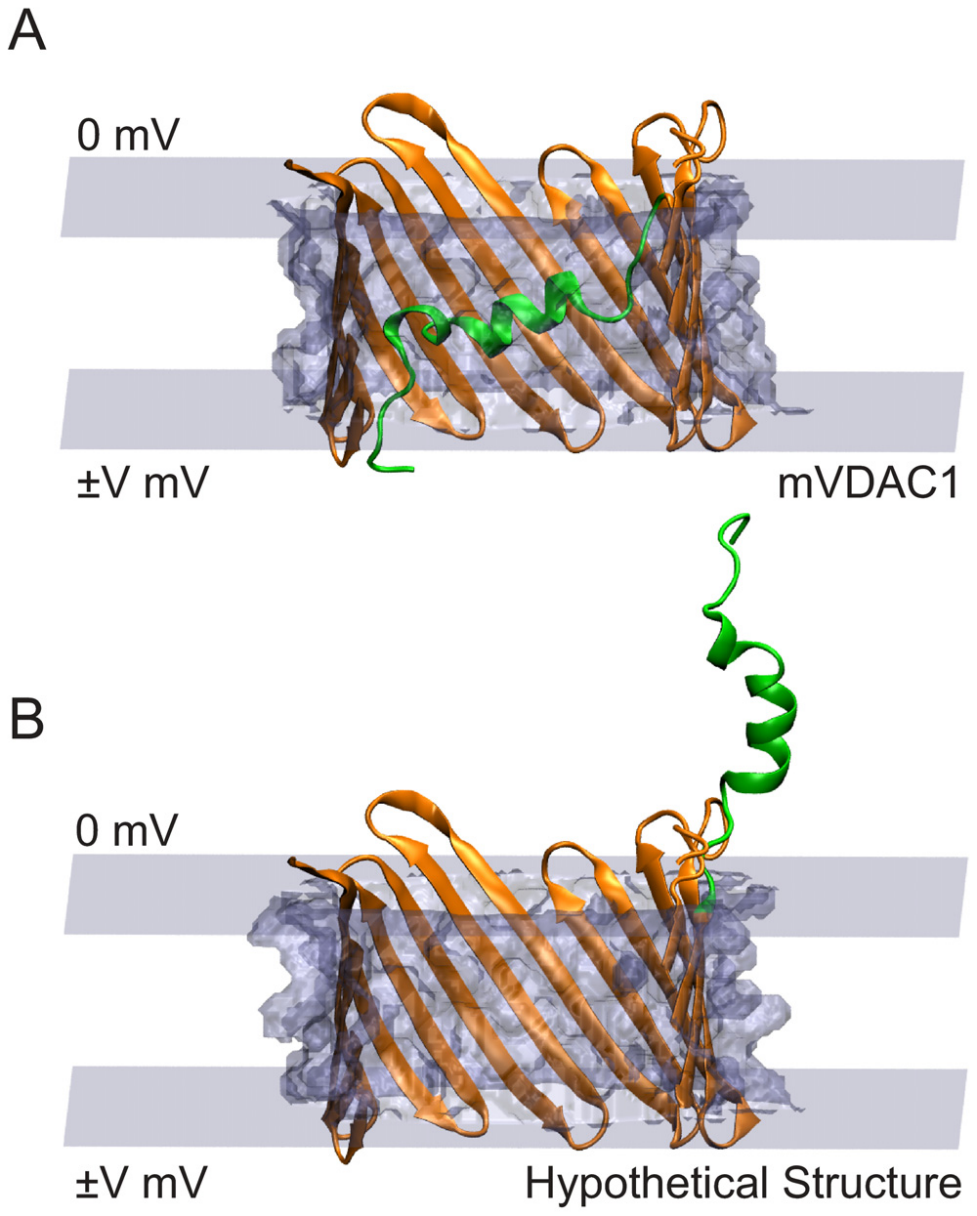
Hypothetical gating motion involving movement of N-terminal helix (green) out of the pore and into the outer bath. (A) mVDAC1 x-ray structure (PDB ID 3EMN) embedded in a slab of low-dielectric material (gray). (B) Hypothetical closed state structure embedded in the membrane. In (A) and (B), the potential at the outer bath is held at 0 mV and the potential at inner bath is varied from −50 mV to +50 mV.

Here we use the murine voltage dependent anion channel 1 (mVDAC1) to illustrate gating charge calculations using APBSmem. The X-ray crystal structure of mVDAC1 shows that it is a 19-stranded 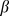-barrel with an N-terminal 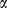-helix thought to be mVDAC1's primary voltage sensor [61]. Both PB and Poisson-Nernst-Planck (PNP) electrostatic calculations on mVDAC1 suggested that the structure represents the open state of the channel [62]. This case study examines the plausibility of a hypothetical gating motion of the channel, ruled out by Choudhary and co-workers [62]. We consider a gating motion in which the N-terminal helix moves out of the channel and into the outer bath, as shown in Figure 7.

The gating charge calculation for this gating motion requires two PQR files - mVDAC1 (PDB ID 3EMN) and a hypothetical closed state structure, to be read in as PQR file 1 and PQR file 2, respectively. We first align mVDAC1 and the hypothetical closed state structure along the z-axis and center them at the origin. The gating charge calculations in this study are carried out on a 161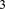 grid with two levels of focusing from a cubic system with side length 300 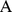 to a smaller cubic system of side length 60 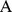. The bathing solution contains 0.1 M symmetric monovalent salt with 2 Å probe radii. The influence of the membrane is included as a dielectric slab of value 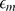 = 2. Water is assigned a dielectric value of 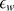 = 80, and the protein dielectric is set to 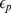 = 5. The head group dielectric (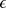 = 80) is only a placeholder variable since its thickness is zero.

The linearized PB equation (lpbe) is solved using focused boundary conditions with one level of focusing at 298.15 K. The interface varies the membrane potential of the inner bath from −50 mV to +50 mV, keeping the potential of the outer bath constant at 0 mV, as shown in Figure 7. A solvent probe radius of 1.4 Å and a surface sphere density of 10 gridpoints/Å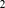 is used. The z-position of the bottom of the membrane and thickness of the membrane slab are set to −14 Å and 28 Å, respectively. The upper and lower exclusion radii for the membrane are both set to 18.5 Å. All the parameters used for this case study are summarized in Table 4.

**Table 4:** Parameters for gating charge calculation CASE III.

Parameter	Value
Calculation type	Gating charge
PQR File 1	3EMN
PQR File 2	Hypothetical closed state
Grid Dimensions	161 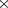 161 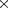 161
Coarse Grid Lengths	300 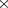 300 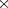 300
Medium Grid Lengths	120 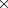 120 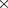 120
Fine Grid Length	60 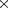 60 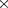 60
Counter-Ions	1.0, 0.10, 2.0
	−1.0, 0.10, 2.0
Protein Dielectric	5.0
Solvent dielectric	80.0
Membrane Dielectric	2.0
Headgroup Dielectric	80.0
Solution Method	lpbe
Boundary Condition	Membrane potential
	(−50 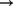 +50 mV)
Solvent probe radius (srad)	1.4
Surface sphere density (sdens)	10.0
Temperature	298.15
Z-position of membrane bottom	−14
Membrane thickness	28
Head group thickness	0
Upper exclusion radius	18.5
Lower exclusion radius	18.5

APBSmem performs PB calculations to determine the membrane potential's contribution to the energy difference between mVDAC1, 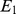, and the hypothetical closed structure, 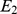 (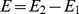). The energy difference is due to interaction of the protein charges with the membrane electric field. Note that the N-terminal helix has a net charge of +2. The slope of the voltage dependence curve is a measure of voltage-sensor valence which is 1.58 e in this case. This value is very close to that obtained by Choudhary and co-workers [62]. These calculations are useful for determining the voltage sensitivity of a proposed gating mechanism, and within 2.5% of the best estimate they converge to a coarse grid of 1 Å (Figure 2). As long as researchers can provide models of hypothetical transitions, these gating calculations can be used to help evaluate their biophysical correctness.

## Discussion

APBSmem is an easy to use software package that carries out electrostatic calculations in the presence of a membrane. We have provided three common cases of interest to researchers in this field. The first calculates the electrostatic penalty of moving charged proteins into the membrane. This has implications for the stability of membrane proteins and for the design of membrane-permeable molecules. The second example examines the electrostatic energy for moving ions through or into ion channels and transporters. Finally, we showed how APBSmem can be used to determine the voltage dependence of a particular molecular movement. As noted earlier in the protein solvation case study, the membrane is modeled as a dielectric slab of variable thickness. Choe *et al.* [53] discussed the significant effects of membrane bending and its relationship to charged particles. APBSmem will eventually be expanded to identify optimal membrane deformations near the embedded molecule to provide a more complete picture of membrane protein energetics.

APBSmem has been tested on Linux, Mac OS X, and Windows, and both source code and binaries are available for download at http://mgrabe1.bio.pitt.edu/apbsmem.
